# Supplementary material for: Displacement of Gray Matter and Incidence of Seizures in Patients with Cerebral Cavernous Malformations
Source: Biomedicines. 2021 Dec 10;9(12):1872. doi: 10.3390/biomedicines9121872 (PMC8698264; doi:10.3390/biomedicines9121872)
Supplement: Supplementary file 1 [file biomedicines-09-01872-s001.zip › biomedicines-1445448-supplementary/biomedicines-1445448 supplementary 1/Supplementary File(s).pdf]

biomedicines

MDPI

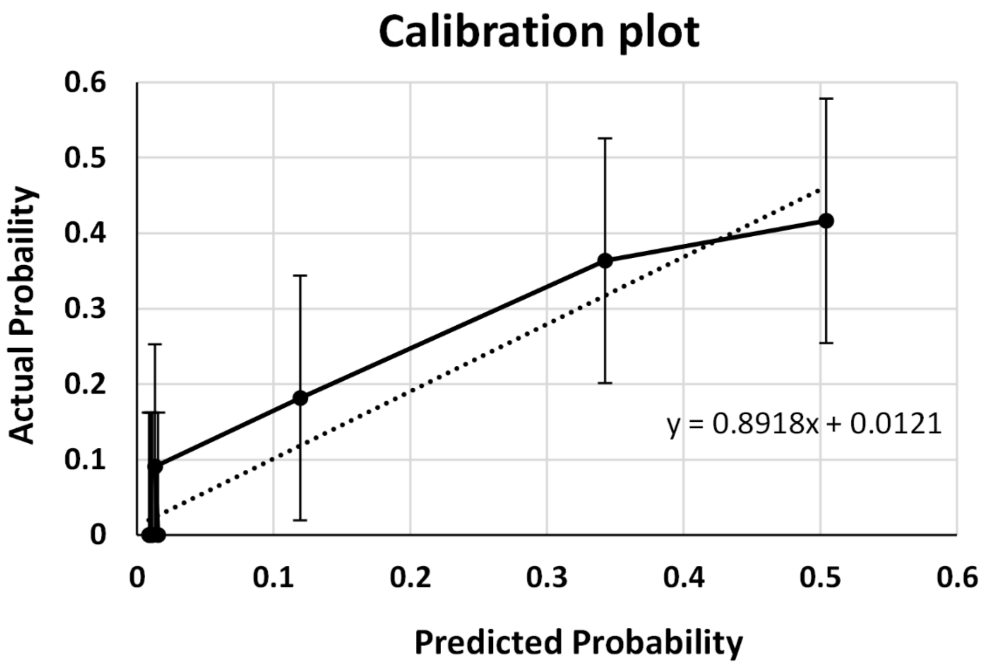

Figure S1. Calibration slope of calibration plot.

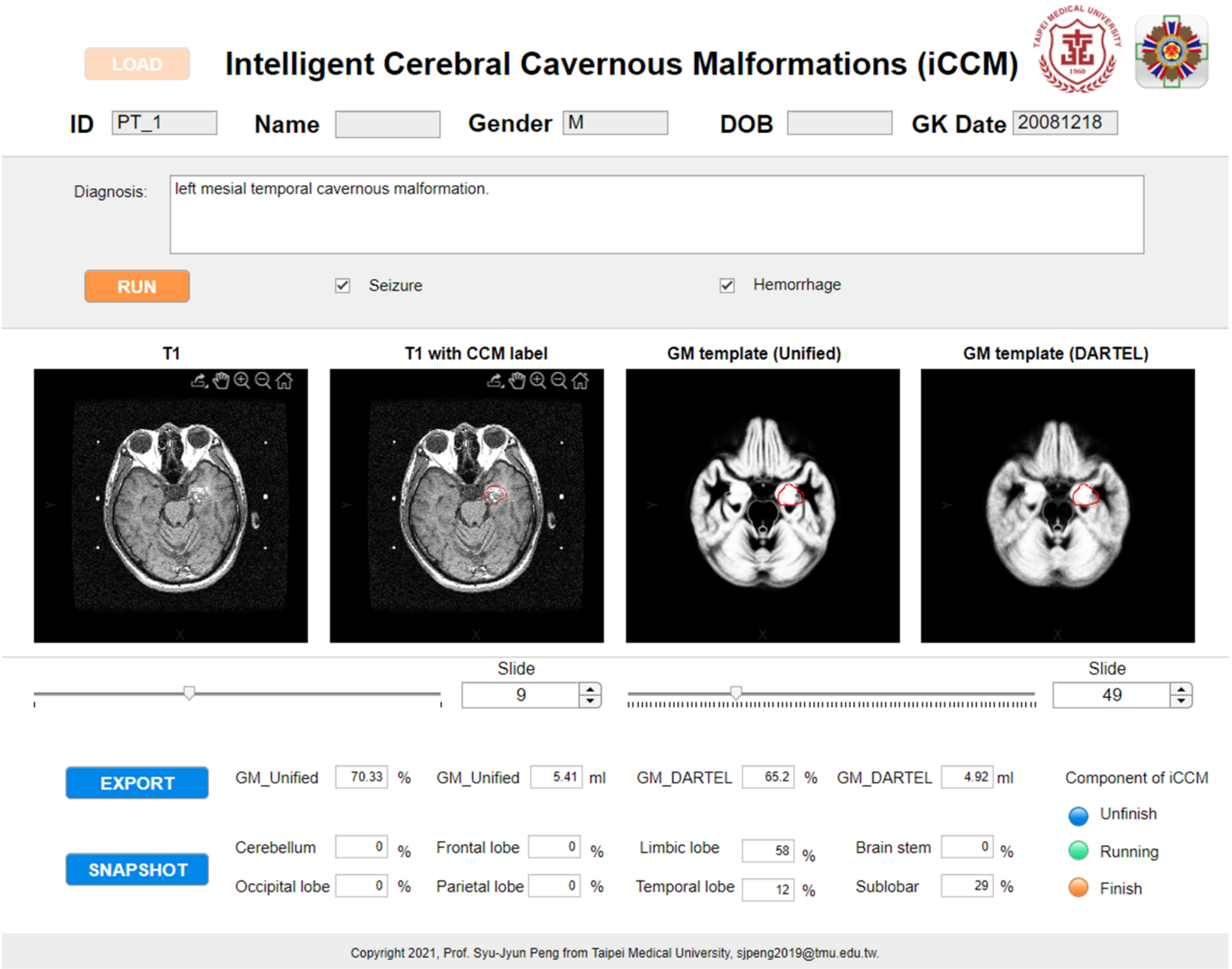

Figure S2. Overview of intelligent cerebral cavernous malformations (iCCM) tool, including LOAD, RUN, EXPORT, and SNAPSHOT functions. LOAD initiates the input of T1WI DICOM data, RUN initiates computer assisted analysis in quantifying the displaced volume and proportion in GM and CCM regions, EXPORT outputs the analysis and results, and SNAPSHOT captures an image of the processed T1WI and iCCM GUI results page.

Table S1. Performance of the discrimination between seizure and nonseizure groups.

Indices	Value	95% CI
AUC	0.901	0.823–0.978
Sensitivity	0.917	0.905–0.929
Specificity	0.838	0.816–0.860
PPV	0.407	0.368–0.446
NPV	0.988	0.986–0.990
F1-score	0.564	0.524–0.604
DOR	0.570	0.530–0.610
MCC	0.546	0.506–0.586
Kappa	0.487	0.446–0.528

AUC: Area Under the Curve; PPV: Positive Predictive Value; NPV: Negative Predictive Value; DOR: diagnostic odds ratio; MCC: Matthew's Correlation Coefficient; Kappa: Kappa coefficient, Confidence intervals (CI).

Table S2. Distribution of CCM as a function of location in the standard brain space.

**Structure**	**Seizure (n=12)** (%)	**Nonseizure (n=99)** (%)	***p***
Frontal lobe	30.427 ± 36.919	10.962 ± 27.475	0.001*
Temporal lobe	15.362 ± 29.146	2.392 ± 12.867	0.002*
Parietal lobe	14.870 ± 32.267	2.106 ± 9.863	0.048*
Occipital lobe	3.648 ± 12.146	0.419 ± 3.997	0.011*
Sublobar	16.673 ± 28.581	15.822 ± 31.238	0.341
Limbic lobe	17.595 ± 20.751	2.804 ± 11.258	<0.001*
Cerebellum	0.210 ± 0.728	11.872 ± 24.337	0.062
Brainstem	0.000 ± 0.000	47.687 ± 41.392	<0.001*

* p value < 0.05 indicating statistical significance via Wilcoxon rank sum test.
